# Proteomic analyses identify prognostic biomarkers for pancreatic ductal adenocarcinoma

**DOI:** 10.18632/oncotarget.23929

**Published:** 2018-01-03

**Authors:** Dingyuan Hu, Daniel Ansari, Krzysztof Pawłowski, Qimin Zhou, Agata Sasor, Charlotte Welinder, Theresa Kristl, Monika Bauden, Melinda Rezeli, Yi Jiang, György Marko-Varga, Roland Andersson

**Affiliations:** ^1^ Lund University, Faculty of Medicine, Department of Clinical Sciences Lund (Surgery), Lund, Sweden; ^2^ Department of Gastroenterology, The Second Affiliated Hospital of Wenzhou Medical University, Wenzhou, China; ^3^ Lund University, Skane University Hospital, Department of Clinical Sciences Lund (Surgery), Lund, Sweden; ^4^ Department of Experimental Design and Bioinformatics, Warsaw University of Life Sciences, Warsaw, Poland; ^5^ Department of Translational Medicine, Lund University, Malmö, Sweden; ^6^ Department of Pathology, Skåne University Hospital, Lund, Sweden; ^7^ Lund University, Department of Clinical Sciences Lund, Division of Oncology and Pathology, Lund, Sweden; ^8^ Department of Biomedical Engineering, Clinical Protein Science and Imaging, Lund University, Lund, Sweden

**Keywords:** pancreatic ductal adenocarcinoma, survival, biomarker, proteome, tumor microenvironment

## Abstract

Pancreatic ductal adenocarcinoma (PDAC) is a highly aggressive malignancy. Here we show that shotgun and targeted protein sequencing can be used to identify potential prognostic biomarkers in formalin-fixed paraffin-embedded specimens from 9 patients with PDAC with “short” survival (<12 months) and 10 patients with “long” survival (>45 months) undergoing surgical resection. A total of 24 and 147 proteins were significantly upregulated [fold change ≥2 or ≤0.5 and P<0.05; or different detection frequencies (≥5 samples)] in patients with “short” survival (including GLUT1) and “long” survival (including C9orf64, FAM96A, CDH1 and CDH17), respectively. STRING analysis of these proteins indicated a tight protein-protein interaction network centered on TP53. Ingenuity pathway analysis linked proteins representing “activated stroma factors” and “basal tumor factors” to poor prognosis of PDAC. It also highlighted TCF1 and CTNNB1 as possible upstream regulators. Further parallel reaction monitoring verified that seven proteins were upregulated in patients with “short” survival (MMP9, CLIC3, MMP8, PRTN3, P4HA2, THBS1 and FN1), while 18 proteins were upregulated in patients with “long” survival, including EPCAM, LGALS4, VIL1, CLCA1 and TPPP3. Thus, we verified 25 protein biomarker candidates for PDAC prognosis at the tissue level. Furthermore, an activated stroma status and protein-protein interactions with TP53 might be linked to poor prognosis of PDAC.

## INTRODUCTION

Pancreatic ductal adenocarcinoma (PDAC) has recently surpassed breast cancer to become the third leading cause of cancer-related mortality according to the American Cancer Society, with a 5-year survival in the single digits [1]. Despite improvements in surgical techniques and adjuvant chemoradiotherapy, the survival from the disease has not changed substantially over the past four decades. It is estimated that PDAC will surpass colorectal cancer to become the second leading cause of cancer-related mortality following lung cancer by the year 2020 [2]. The main reason underlying the low survival rate of PDAC is that most patients are diagnosed at an advanced stage, at which curatively intended surgery, no longer represents an option. Currently, CA19-9 is the only serum tumor marker used in the clinical management of PDAC. However, the sensitivity for CA19-9 is 79% with a specificity of 82%, limiting its use for screening purposes [3].

Traditionally, PDAC has been looked upon as a gradual process associated with the sequential accumulation of genetic changes during a comparably long period of time [4]. Novel data has though implied that the development of PDAC may not be a slow and gradual process. Using whole genome sequencing, it was reported that genomic instability from mitotic errors might occur simultaneously resulting in rapid tumor development and metastases in a subset of patients [5]. These findings have been supported by a recent publication on approximately 60,000 patients with histopathologically verified PDAC where survival and metastatic spread were correlated to tumor size [6]. It was reported that already at a small tumor size up to 5 mm, as much 30% of patients had remote cancer growth. This implies the predominant role of molecular tumor biology in determining outcome for the individual patient. It also emphasizes the need for better tools for staging, for example with novel biomarkers in order to render the necessary prognostic and predictive information and support choice of therapy in a more precision-medicine fashion.

While large scale genomics studies have provided understanding of mutational processes underlying the development of PDAC [7, 8], and helped to define molecular subtypes of PDAC [9, 10], proteomics technology has accelerated our understanding of PDAC at the protein level by identifying key drivers of disease progression and biomarkers for diagnosis and targeted intervention [11, 12]. Recent proteomic studies and further validation studies have greatly expanded the pool of potential diagnostic and prognostic biomarkers in PDAC. For instance, at the tissue level, Turtoi et al. found that ASPN, LTBP2, TGFBI were overexpressed in PDAC [13], while Takadate and colleagues suggested that ECH1, GLUT1, OLFM4 and STML2 were potentially diagnostic biomarkers of PDAC [14]. Furthermore, Chen and colleagues found that PRELP, LGALS1 and RPS8 might be significant prognostic factors for pancreatic cancer [15], while another study showed that PNMA1 was associated with prolonged overall survival and might serve as a prognostic biomarker for pancreatic cancer [16]. At the plasma level, ICAM1 and TIMP1 have been proposed as biomarkers for the detection of pancreatic cancer [17]. However, these biomarkers were mostly studied in small population cohorts and thus further validation is warranted prior to clinical use.

Formalin-fixed paraffin-embedded (FFPE) tissues are used routinely in hospitals for histopathological diagnosis and staging of diseases like cancer. FFPE samples with associated clinical and histological characterization represent a valuable source of biomarker investigation. The application of mass spectrometry technology to FFPE samples has been shown to be technically feasible and highly robust for biomarker discovery and validation [18]. Specifically, deep mining of proteomes from individual samples, including membrane proteins and low-abundance proteins, broadens the possibility to discover potential biomarkers. Using proteome bioinformatic tools, the many functional partnerships and interactions that occur between proteins are revealed and put into context for molecular systems biology. In our study, we selected tissue samples from PDAC patients with divergent survival, aiming to identify prognostic biomarker panels correlating with outcome.

## RESULTS

### Quality control and overview of proteome profiles

**Figure 1:**
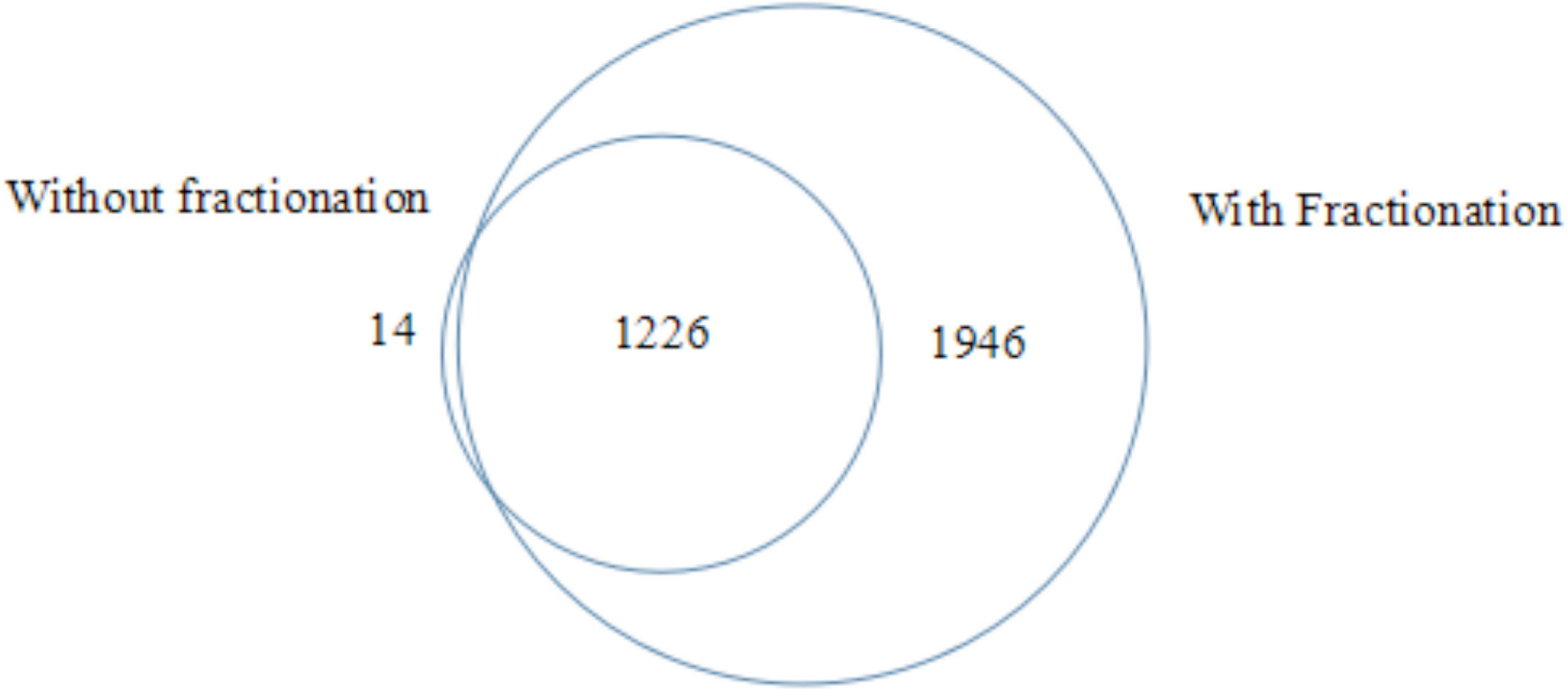
Venn diagram of protein numbers being identified in one identical sample by methods with and without fractionation

To evaluate the technical reproducibility of sample handling including reduction, alkylation, precipitation and fractionation and instrument performance, we performed three independent sample preparations using an identical protein stock extracted from one sample. The intensities of proteins in the three experiments showed good correlations (r^2^ = 0.973, 0.920 and 0.931). Besides, sample preparations with and without fractionation were applied to an identical sample to compare the consistency of protein intensities from these two methods. In result, the protein intensities were in good correlation between the two methods (r^2^ = 0.9373). Moreover, the fractionation step achieved a remarkable enlargement of protein number being identified, which enabled a deep mining of proteome in pancreatic tissue in this study (Figure 1). Among 57 replicates from 19 samples, coefficient of variations (CV) of Log 2 transformed intensities of spiked-in chick lysozyme before and after normalization was 34.0% and 6.6%, respectively. Around 3,000,000 peptide-spectrum matches (PSMs) and 58,505 peptides with high confidence were identified, which were mapped to 4942 proteins (minimum 2 peptides per protein). Among them, 3103 proteins were identified in more than half (≥ 5) of the samples in at least one group. Gene Ontology analysis was conducted based on the 3103 proteins. Cellular component analysis showed that there were 640 plasma membrane proteins, 108 cell surface proteins and 163 extracellular matrix proteins, which were considered as potential proteins for potential serum detection and also candidate therapeutic targets. Notably, PANTHER pathway analysis indicates that Integrin signaling pathway is significantly enriched (3.23 fold, *P* = 6.58E-19).

### Candidate prognostic proteins for PDAC

**Figure 2:**
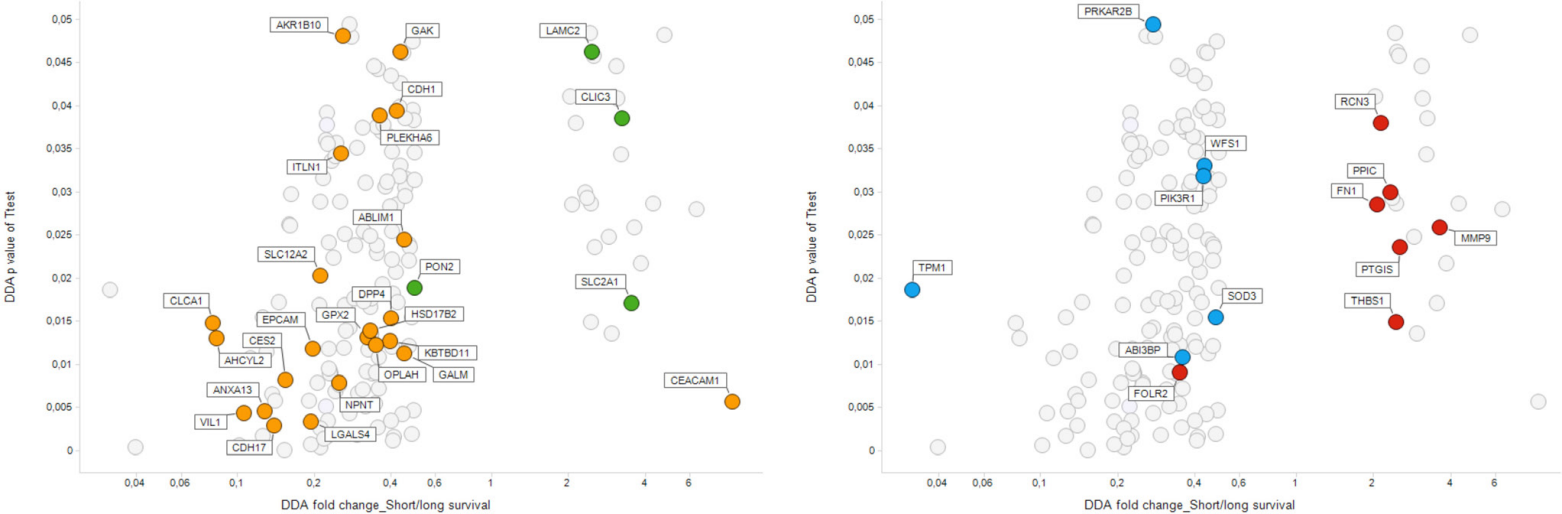
Volcano plot Comparison of protein expression in short survival tumors (SS) vs long survival ones (LS). Vertical axis: *t*-test *p*-value, horizontal axis: SS/LS fold change. Colouring by proteins characteristic for PDAC subtype factors according to Moffitt et al. Left: Tumor-related factors: Green: Basal tumor, Orange: Classic tumor. Right: Stroma related-factors. Blue: Normal stroma, Red: Activated stroma. (see text).

A total of 304 proteins were differentially expressed between the “long” survival (LS) and “short” survival (SS) groups (*P* < 0.05), including 33 proteins and 271 proteins statistically upregulated in “short” survival group and “long” survival group, respectively. Among them, 171 proteins were significantly differentially expressed between the two groups which meet the criteria: 1) SS/LS fold change ≥ 2 or ≤ 0.5 and *P* < 0.05; or 2) different detection frequencies (≥ 5 samples), namely, 83 proteins that were more frequently detected (≥ 5) in one group than the other one. Of these 171 proteins, 24 and 147 proteins were upregulated in “short” survival group and “long” survival group, respectively (see Figure 2).

**Figure 3:**
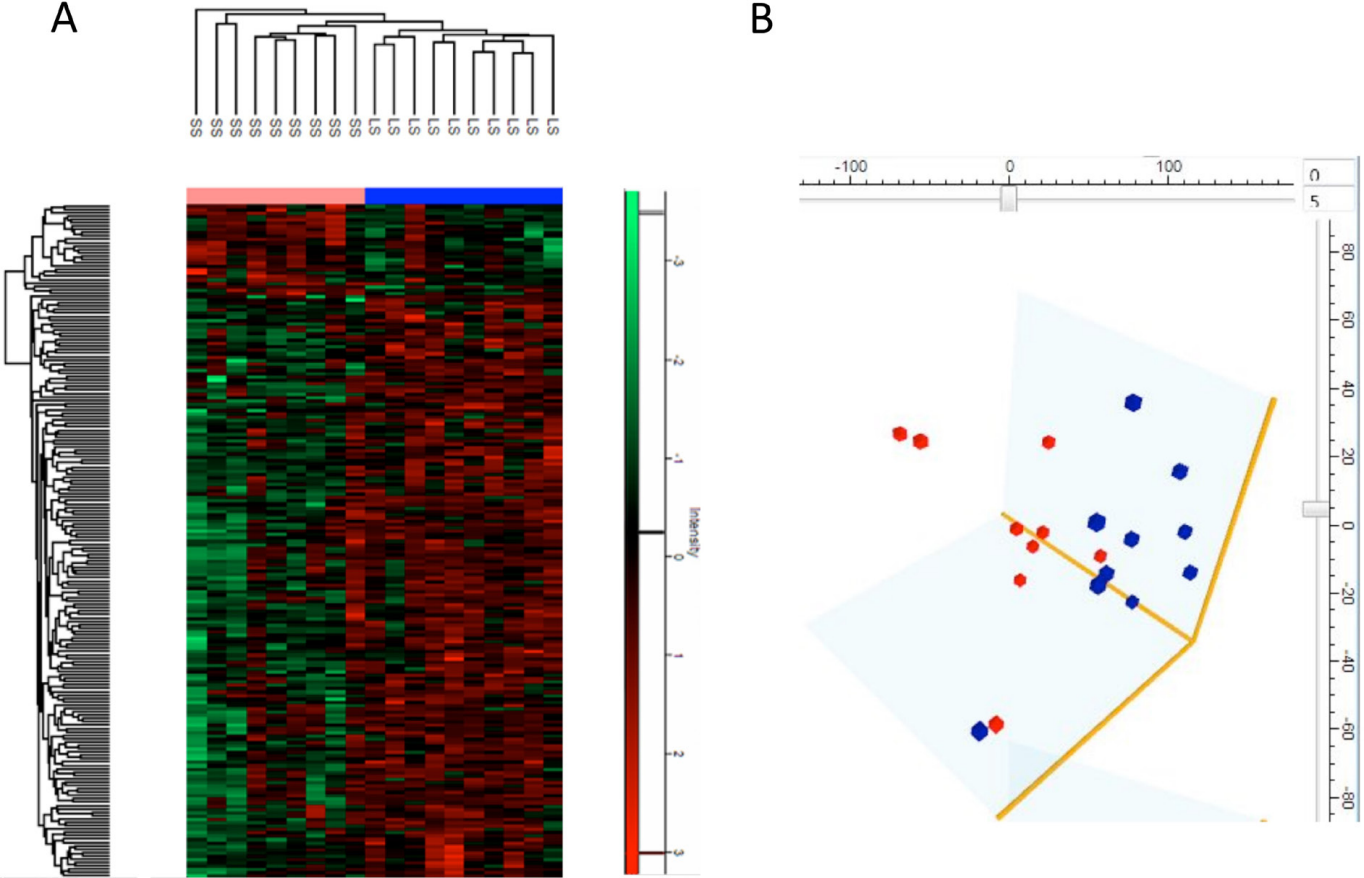
(**A**) Heat map of differentially expressed proteins in pancreatic cancer with long survival (LS) and short survival (SS). The heat map visualized two-way unsupervised hierarchical clustering of 171 differentially expressed proteins in pancreatic cancer patients with short survival (SS) compared to those with long survival (LS) (*P* < 0.01, SS/LS fold Change ≥ 2). (**B**) Global principal component analysis of protein profiles in 19 samples. Dots representing pancreatic cancer (PC) patient samples with long survival (blue) and short survival (red) were well clustered, which was in good agreement with the clinical classification.

The 171 differentially expressed proteins from 19 tissue samples were submitted to two-way unsupervised hierarchical clustering and visualized in the heat map (Figure 3A). The clustering of 19 tissue samples was in good agreement with the clinical classification. The principal component analysis further confirmed that patients with “long” survival and “short” survival were well stratified by group of differentially expressed proteins (Figure 3B). The set of differentially expressed proteins exhibited striking trend in terms of subcellular localization. David analysis showed significant overrepresentation of mitochondrial proteins (34 proteins, *P*-value 0.017), and specifically mitochondrial large ribosomal subunit (6 proteins, *P*-value 0.002) and mitochondrial respiratory chain complex I (5 proteins, *P*-value 0.033). PANTHER pathways analysis of differentially expressed proteins revealed overrepresentation of Wnt signaling pathway (CDH1, CSNK2A2, GNA11, CTBP2, CDH17, SMARCE1, *p-value* 0.02), followed by Alzheimer disease-presenilin pathway (MMP8, MMP9, MLLT4, CDH1, *P*-value 0.04).

In order to better assess proteins upregulated in “short” and “long” survival groups, these two sets (24 and 147 proteins, respectively), were separately submitted to Panther functional analysis. Among proteins upregulated in the “long” survival group, remarkably overrepresented were mitochondrial proteins (*P*-value 3e-5), which translated into overrepresentation of oxidoreductase activity (*P*-value 1.8e-3). Among proteins upregulated in the “short” survival group, overrepresented were secretory vesicle proteins (*P*-value 5e-6) and extracellular proteins (*P*-value 4e-4). This was related to overrepresentation of activities such as peptidase activity (*P*-value 2.5e-2), collagen binding (*P*-value 4.8e-4), heparin binding (*P*-value 7e-6) and lipid binding (*P*-value 3e-2).

**Figure 4:**
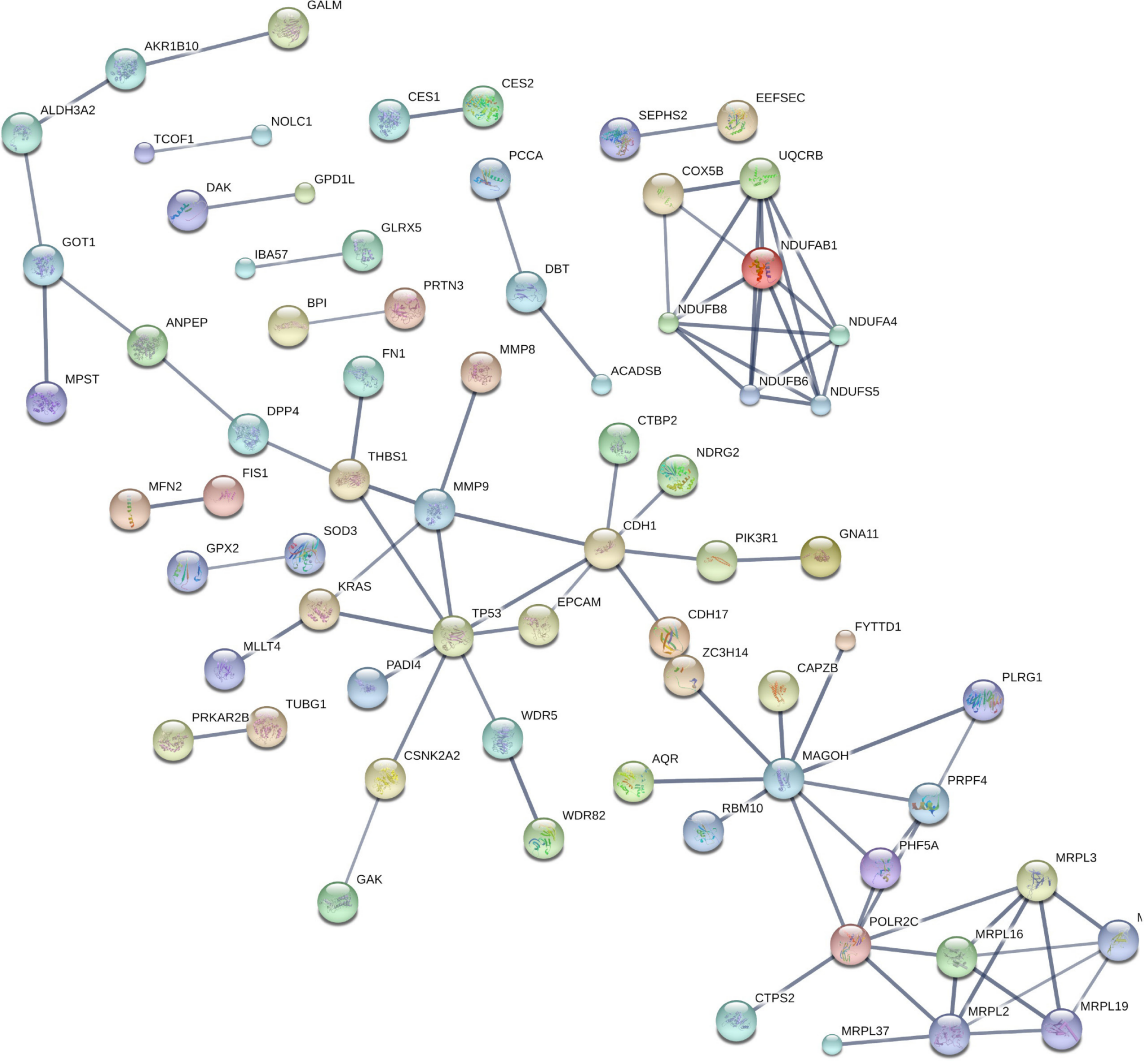
Protein-protein interactions among prognostic candidate proteins Protein-protein interactions of the 171 dysregulated proteins extracted from the STRING database. TP53, KRAS were manually added to identify potentially related pathways. Notably, seven proteins were centered on TP53, including CDH1, THBS1, MMP9, EPCAM, WDR5, CSNK2A2, PADI4.

STRING database [19] was employed to investigate the functional and physical protein interactions among the 171 differentially expressed proteins (Figure 4). Since TP53 and KRAS were essential in the pathogenesis of pancreatic cancer, these two proteins were manually added to identify potentially related pathways. With high confidence (minimum required interaction score 0.700), a total of 86 protein-protein interactions were observed and they were significantly enriched based on the given protein nodes (*P*-value < 0.001), indicating that these differentially expressed proteins are at least partially biologically connected. Seven proteins clustered in a tight interaction network centered on TP53, including CDH1, THBS1, MMP9, EPCAM, WDR5, CSNK2A2, PADI4. Of this protein cluster, CDH1 also closely interacts with CDH17, PIK3R1, NDRG2, CTBP2, MMP9 and EPCAM while THBS1 is centered by FN1, DPP4 and MMP9. Besides, intensive interactions were also observed in the other three clusters of proteins, which were related to respiratory electron transport (COX5B, UQCRB, NDUFS5, NDUFA4, NDUFAB1, NDUFB6 and NDUFB8), mitochondrial translation (MRPL37, MRPL2, MRPL3, MRPL16, MRPL19 and MRPL23) and mRNA Splicing (PRPF4, POLR2C, MAGOH, PLRG1, CWC15 and PHF5A).

**Figure 5:**
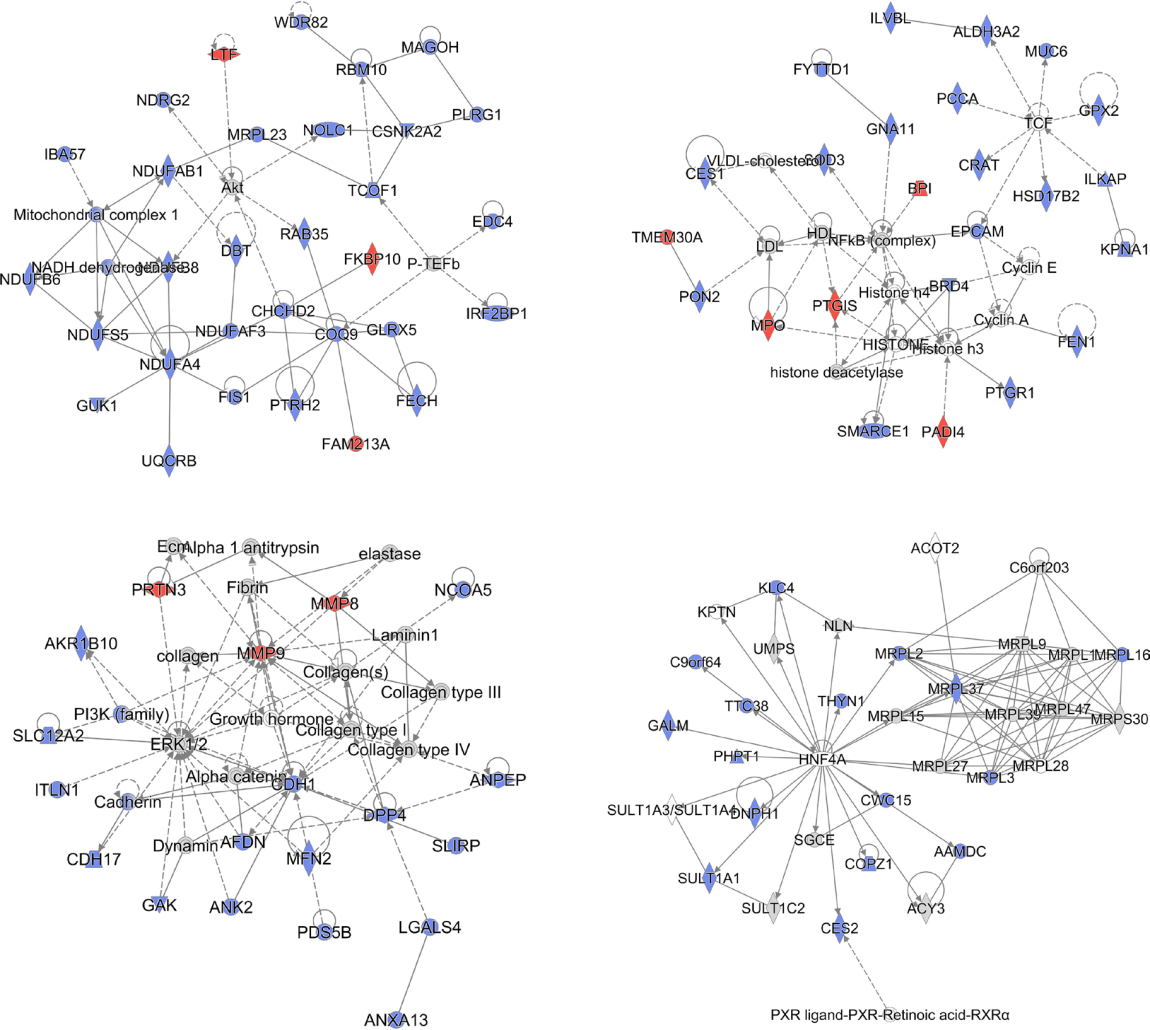
Protein-protein relationships among prognostic candidate proteins extracted by the Ingenuity IPA analysis Top four subnetworks shown. Red: proteins upregulated in short survival patients. Blue: proteins upregulated in long survival patients.

A complementary Ingenuity Pathway Analysis (IPA), using curated, literature-derived relationships, showed a picture similar to the STRING analysis (Figure 5). Top canonical pathways, which were significantly enriched among proteins differing between the “short” survival group and “long” survival group, included Oxidative Phosphorylation and Mitochondrial Dysfunction. For example, the differentially expressed proteins amounted to 7 out of 22 Oxidative Phosphorylation pathway proteins (*P*-value 0.002). Similarly to the non-curated networks generated by STRING, also Ingenuity analysis yielded tightly connected relationship subnetworks, built around protein hubs, which are known PDAC actors, even if these hub proteins were not themselves differentially expressed. These subnetworks are constructed automatically as dense subsets of global network of literature-derived relationships between proteins and genes. First such subnetwork was centered on Akt kinase and mitochondrial complex 1 proteins. The second subnetwork was centered on NFkB and TCF transcription factors. The hubs of the third subnetwork were the ERK kinases, collagens and matrix metalloproteases (MMPs). The fourth subnetwork was focused on HNF4A and mitochondrial ribosomal proteins.

**Figure 6:**
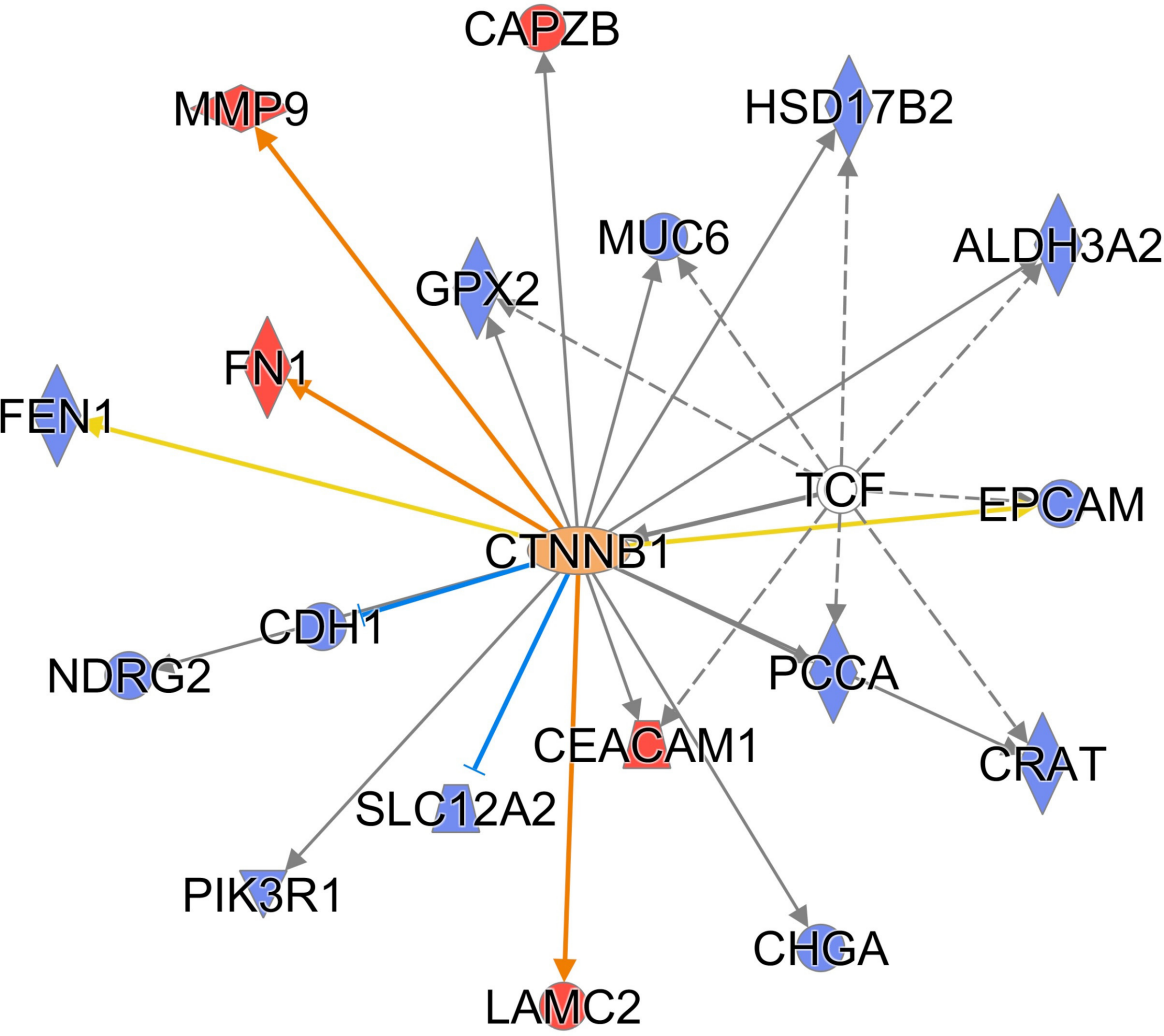
TCF1 and CTNNB1 are hubs of a mechanistic upstream regulatory network (IPA) Red symbols: proteins upregulated in short survival patients. Blue symbols: proteins upregulated in long survival patients. Orange edges: relationships predicted as activating. Blue edges: relationships predicted as inhibitory. Yellow edges: relationships inconsistent with downstream protein state.

Additionally, an IPA analysis of possible upstream regulators of the differentially expressed proteins yielded a mechanistic network regulated by HNF1A (TCF1) and CTNNB1, a well-known cancer regulatory hub important for the Wnt signaling pathway. The HNF1A mechanistic network was significant, with *p*-value 4.2E-05, and included 8 proteins from the differentially expressed list: ALDH3A2, CEACAM1, CRAT, EPCAM, GPX2, HSD17B2, MUC6 and PCCA, see Figure 6.

### Verification of candidate prognostic proteins by targeted MS/MS

**Figure 7:**
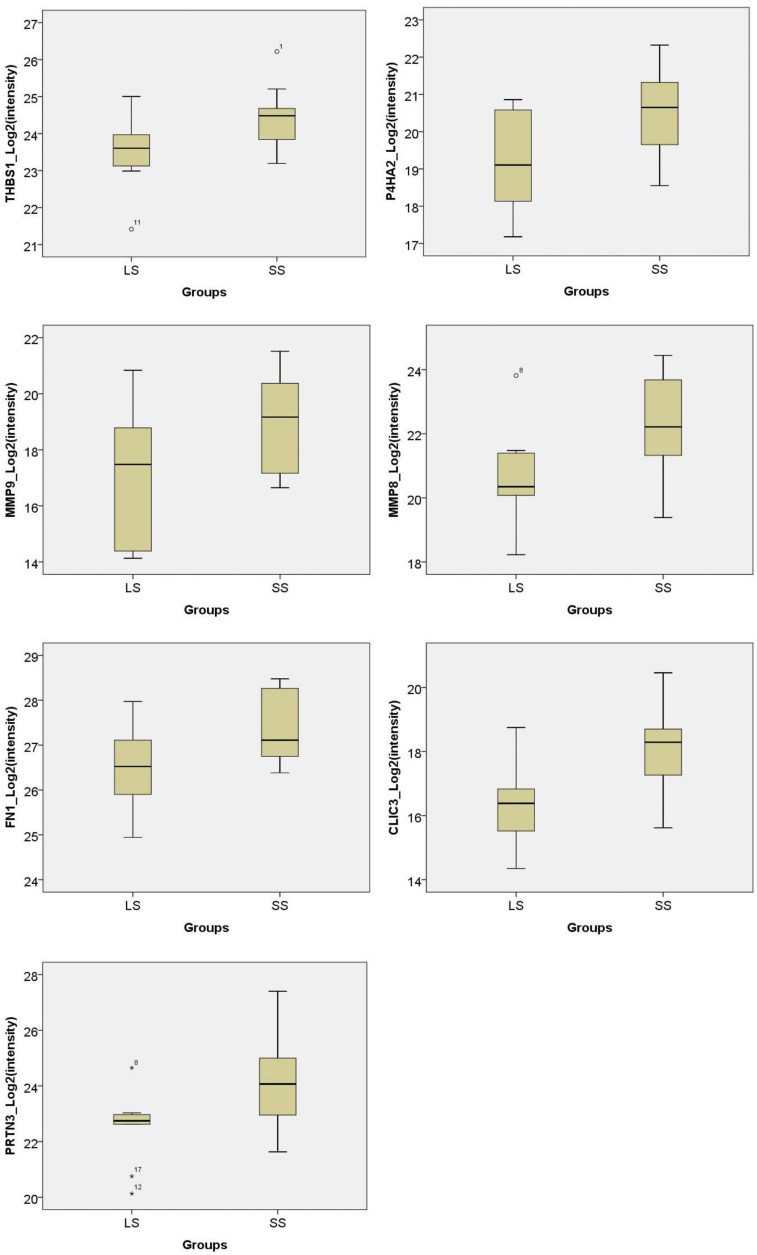
Boxplot of intensities of prognostic proteins from PRM phase in PDAC patients with “short” survival (SS) compared to “long” survival (LS) (all *P <* 0.05) Seven proteins (THBS1, P4HA2, MMP9, MMP8, FN1, CLIC3, PRTN3) were significantly upregulated (SS/LS fold change > 1.5) in patients with SS while CLCA1 were significantly upregulated (SS/LS fold change < 0.5) in patients with LS.

**Figure 8:**
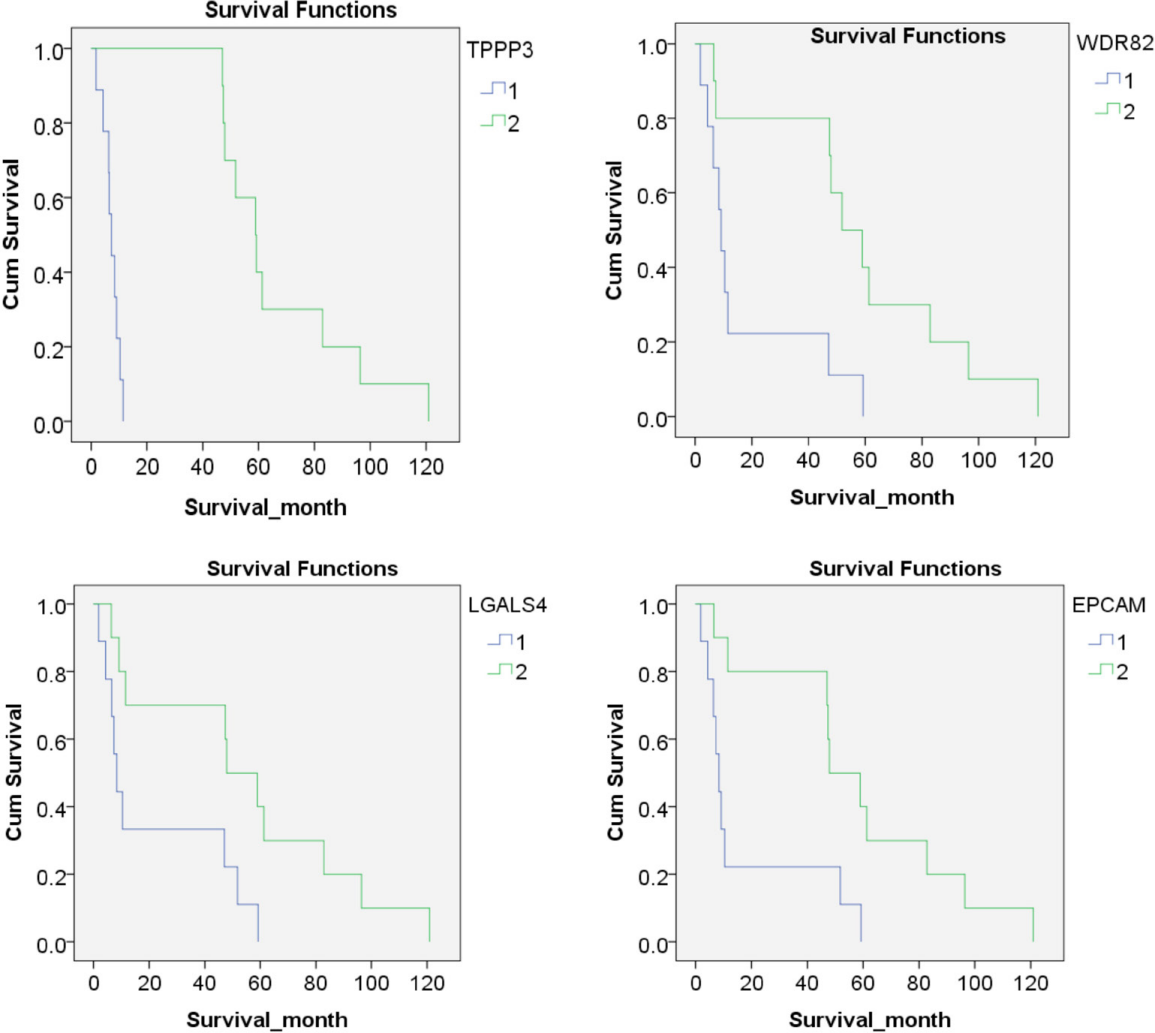
Kaplan-Meier analysis of protein expression According to the expression of each protein, patients were divided into two groups: lower expression (9 cases, Blue line, marked by 1) and higher expression (10 cases, green line, marked by 2), Kaplan-Meier analysis showed that four proteins were significantly correlated to the survival months (TPPP3, WDR82, LGALS4 and EPCAM, *P* values were < 0.001, 0.008, 0.020 and 0.010, respectively).

To evaluate the potential candidate proteins, 171 differentially expressed proteins from the discovery phase were selected for targeted proteomics study. Unfortunately, 98 proteins of them failed in the PRM approach. Finally, 73 proteins were successfully detected and scheduled in one assay panel. The proteins were detectable in all samples. Thirty-six proteins were differentially expressed between the two groups, including 7 proteins and 29 proteins statistically upregulated in “short” survival group and “long” survival group, respectively (*P* < 0.05). Of them, seven proteins were significantly upregulated (SS/LS fold change > 1.5) in patients with “short” survival (MMP9, CLIC3, MMP8, PRTN3, P4HA2, THBS1, FN1), while 18 proteins were significantly upregulated (SS/LS fold change <0.5) in patients with “long” survival (TMED4, GPD1L, SOD3, NPNT, ABHD14B, ACADSB, DHRS1, EPCAM, WDR82, HDHD2, TPPP3, CHGA, LGALS4, TTC38, COQ9, CES2, VIL1, CLCA1) (Figure 7 and Table 1). After the expression values of each protein were divided into two groups: lower expression (9 cases) and higher expression (10 cases), Kaplan-Meier analysis showed that four proteins were significantly negatively correlated to the survival months (TPPP3, WDR82, LGALS4 and EPCAM, *P* values were < 0.001, 0.008, 0.020 and 0.010, respectively) (Figure 8).

**Table 1:** List of candidate prognostic biomarkers for pancreatic cancer

Entry	Gene	DDA				PRM			Description
		LS Freq.	SS Freq.	*P* value	SS/LS Fold change	Pep. no.	*P* value	SS/LS Fold change	
P14780	MMP9	10	9	0.026	3.62	2	0.045	4.44	Matrix metalloproteinase-9
O95833	CLIC3	1	6	0.039	3.25	2	0.010	3.42	Chloride intracellular channel protein 3
P22894	MMP8	1	6	0.029	4.28	1	0.046	3.06	Neutrophil collagenase
P24158	PRTN3	7	9	0.022	3.85	2	0.031	2.98	Myeloblastin
O15460-2	P4HA2	6	9	0.025	2.88	2	0.029	2.66	Isoform IIa of Prolyl 4-hydroxylase subunit alpha-2
P07996	THBS1	10	9	0.015	2.46	2	0.028	2.01	Thrombospondin-1
P02751	FN1	10	9	0.029	2.07	2	0.034	1.92	Fibronectin
A8K7I4	CLCA1	5	0	0.015	0.08	1	0.029	0.05	Calcium-activated chloride channel regulator 1
P09327	VIL1	10	5	0.004	0.11	2	0.008	0.12	Villin-1
O00748	CES2	5	0	0.008	0.15	2	0.029	0.16	Cocaine esterase
O75208	COQ9	8	2	0.004	0.27	2	0.004	0.19	Ubiquinone biosynthesis protein COQ9, mitochondrial
Q5R3I4	TTC38	10	5	0.017	0.21	1	0.035	0.20	Tetratricopeptide repeat protein 38
P56470	LGALS4	10	8	0.003	0.19	2	0.005	0.23	Galectin-4
P10645	CHGA	7	2	0.038	0.23	2	0.025	0.27	Chromogranin-A
Q9BW30	TPPP3	9	1	0.001	0.10	1	0.000	0.28	Tubulin polymerization-promoting protein family member 3
Q9H0R4	HDHD2	10	5	0.005	0.22	2	0.026	0.30	Isoform 2 of Haloacid dehalogenase-like hydrolase domain-containing protein 2
Q6UXN9	WDR82	7	2	0.039	0.22	1	0.023	0.31	WD repeat-containing protein 82
P16422	EPCAM	8	2	0.012	0.20	1	0.021	0.33	Epithelial cell adhesion molecule
Q96LJ7	DHRS1	7	1	0.007	0.24	1	0.017	0.37	Dehydrogenase/reductase SDR family member 1
P45954	ACADSB	9	3	0.017	0.15	2	0.026	0.38	Short/branched chain specific acyl-CoA dehydrogenase, mitochondrial
Q96IU4	ABHD14B	10	9	0.007	0.31	2	0.013	0.46	Alpha/beta hydrolase domain-containing protein 14B
Q6UXI9-6	NPNT	10	3	0.008	0.25	1	0.016	0.46	Isoform 6 of Nephronectin
P08294	SOD3	10	9	0.015	0.48	2	0.003	0.47	Extracellular superoxide dismutase [Cu-Zn]
Q8N335	GPD1L	9	4	0.030	0.16	2	0.010	0.47	Glycerol-3-phosphate dehydrogenase 1-like protein
Q7Z7H5	TMED4	10	4	0.002	0.13	2	0.040	0.48	Transmembrane emp24 domain-containing protein 4

Abbreviations: DDA:data-dependent acquisition; PRM:parallel reaction monitoring; LS: long survival; SS: short survival; Pep. No: number of peptides for proteins in the PRM panel. Freq.: number (frequency) of cases in which the protein was detected.

## DISCUSSION

PDAC is considered one of the most aggressive and lethal forms of human cancer. However, there exists a small proportion of patients that actually reach a comparably “long” survival after surgical resection and adjuvant chemotherapy, even when they have “advanced stage” disease (size) or other markers of poor prognosis [20–22]. There have been very few studies relating global protein expression to survival in PDAC [14, 23]. The characterization of protein profiles at the tissue level might help to understand better the molecular basis of PDAC progression and identify potential biomarkers for diagnosis and prognosis of the disease. In this study, we have established a comprehensive method for proteome deep mining based on formalin-fixed paraffin-embedded PDAC tissues, which led to discovery of around 5000 proteins, making it possible to detect low abundance proteins and hydrophobic membrane proteins. A total of 171 proteins were dysregulated in patients with “short” survival compared to those with “long” survival. A further validation panel, targeting 73 of the differentially expressed proteins confirmed that 7 and 18 proteins, were upregulated in the “short” survival and the “long” survival patients, respectively.

In this study, several aspects accounting for the aggressiveness of PDAC have been highlighted. Among well-known hallmarks of cancer metabolism, shift from oxidative phosphorylation towards glycolysis is well-known [24], and specific glucose metabolic phenotype was proposed for pancreatic cancer [25]. Strikingly, in the current study, this metabolic shift was seen as generally lower expression of mitochondrial proteins in “short” survivors. Most notably, this affected a set of mitochondrial respiratory chain complex I proteins (likely resulting in lowered oxidative phosphorylation) and a set of mitochondrial ribosomal proteins (likely resulting in lowered mitochondrial translation rates).

Another cancer metabolism hallmark is the deregulation of glucose intake [24]. Glucose transporter 1 (GLUT1), also known as facilitated glucose transporter member 1 (SLC2A1), is a pivotal rate-limiting element in the transport of glucose in malignant cells. GLUT1 has also been implicated in the pathogenesis of PDAC. Nagarajan et al. found that by stimulating GLUT1-mediated glucose transport, paraoxonase 2 favored the tumor growth and metastasis of PDAC [26]. It has also been reported that HMGB2 predicts poor prognosis in PDAC by facilitating HIF1-α-mediated glycolysis through the expression of GLUT1 [27]. NDRG1, a tumor suppressor, was also shown to inhibit cancer metabolism in PDAC partly through the regulation of GLUT1 gene [28]. High levels of GLUT1 have been previously correlated to poor outcome in PDAC [29, 30]. Accordingly, in our results, GLUT1 was significantly upregulated in “short” survivors. Recently, GLUT1 was shown to be a promising target in pancreatic cancer stem cells in mice [31].

Several differentially expressed proteins (CDH1, THBS1, MMP9, EPCAM, WDR5, CSNK2A2 and PADI4) have a close interplay with TP53, which is frequently mutated and progressively involved in pancreatic cancer [32–35]. THBS1, MMP9 and PADI4 were upregulated in patients with “short” survival, while the other four proteins were upregulated in patients with “long” survival. The predictive potential of THBS1 and MMP9 for the prognosis of pancreatic cancer has been reported in a few previous studies [14, 36, 37]. It has been suggested that TP53 inhibits angiogenesis by the regulation of THBS1 synthesis [38], while MMP9 degrades the extracellular matrix component and facilitates the invasion of tumors. PADI4 acts as a transcriptional corepressor for TP53 [39]. A study revealed that the TP53-PADI4 pathway participated in the response to DNA damage, nuclear fragmentation and TP53-mediated cell death [40]. Inhibition of TP53 was also implicated in the downregulation of CDH1 and cell invasion in invasive carcinoma [41]. Notably, CDH1 has functional protein associations with differentially expressed proteins in our study including CDH17, PIK3R1, NDRG2, CTBP2, MMP9 and EPCAM according to the STRING database. Kaplan-Meier analysis showed that the expression of EPCAM was inversely correlated to the survival (months) of pancreatic cancer. It has been found that the TP53 protein negatively regulates EPCAM expression by binding to a response element within the EPCAM gene [42]. Higher expression of EPCAM is associated with an improved outcome in pancreatic cancer by suppressing cell activity [43, 44].

A histological hallmark of PDAC is that tumor cells are surrounded by as much as 90% stroma consisting of proliferating myofibroblast-like cells (pancreatic stellate cells), immune cells and inflammatory cells and extracellular matrix components such as collagen, fibrinogen, hyaluronan, and fibrin [45]. The microenvironment of pancreatic adenocarcinoma has a complex role in tumor growth and therapeutic response. While the existence of a dense stroma is thought to promote tumor progression and metastasis [46, 47], this concept has been challenged by recent experimental evidence showing that some elements of the stroma may actually restrain the tumor arguing for stromal re-shaping rather than pure depletion [48–50]. A number of clinical trials targeting the tumor-stroma interactions in PDAC are ongoing, however, the results seem to be inconclusive. Therefore, a further understanding of the tumor microenvironment is needed. A recent large-scale genomics analysis of PDAC by Moffitt et al. employed so-called virtual microdissection to elucidate tumor subtypes and to account for cellular heterogeneity in tumor samples, typically containing a large amount of stroma alongside the tumor itself [10]. They linked poor prognosis to sets of proteins named “activated stroma factors” as well as “basal tumor factors”. Strikingly, our data parallels closely to their results. As seen in Figure 2, proteins classified by Moffitt as “activated stroma factors” and “basal tumor factors” were upregulated in short survival patients while proteins classified as “normal stroma factors” and “classic tumor factors” were upregulated in long survival patients. The proteins characteristic for these tumor features made up as much as approximately 20% of differentially expressed proteins. Our results are in accordance with the findings of Moffitt et al. and support the idea that an activated stroma state may be linked to poor prognosis [10].

In our study, many potentially prognostic proteins are related to the microenvironment of pancreatic cancer. Reactome pathway analysis revealed that 9 of the differentially expressed proteins were involved in extracellular matrix organization, including THBS1, PLOD1, LAMC2, P4HA2, MMP9, MMP8, FN1, CDH1 and CEACAM1. Four proteins participating in collagen formation, PLOD1, LAMC2, P4HA2 and MMP9, were all upregulated in patients with “short” survival compared to those with “long” survival. In comparison, out of five proteins participating in degradation of the extracellular matrix, four (LAMC2, FN1, MMP8 and MMP9) were upregulated and one (CDH1) was downregulated in the poor outcome group. This to some extent again suggests that the microenvironment in “short” survival patients was more activated, both in the formation and degradation of the extracellular matrix, which is believed to provide support to the surrounding tissues and serve as a physical barrier to drug delivery in PDAC [51]. Our study also revealed several collagen associated proteins as potential prognostic biomarkers, including P4HA2, THBS1 and FN1. P4HA2 participates in the biosynthesis of collagens by catalyzing the post-translational formation of 4-hydroxyproline in -Xaa-Pro-Gly- sequences in collagens. Studies have shown that the expression of P4HA2 were upregulated in the oral cavity in squamous cell carcinoma, papillary thyroid cancer, and breast cancer [52]. Furthermore, silencing P4HA2 or treatment with the P4HA inhibitor suppresses breast cancer progression by reducing tumor growth and a metastasis, which is accompanied by reduced collagen deposition, indicating its potential role as therapeutic target. FN1 has been suggested as a prognostic biomarker for pancreatic cancer in a proteomics study [14]. FN1 binds to its receptors such as integrins, inducing distinct signals to promote tumor angiogenesis and migration of PDAC cells [53]. A related molecule, regulator of integrin recycling, the CLIC3 intracellular chloride channel which drives invasiveness of pancreatic cancer is also upregulated in “short” survivors in the current study [54].

Two upstream regulators identified in our prognostic study, TCF1 and CTNNB1, emphasized the potential role of Wnt signaling pathway whose improper activation is responsible for establishment of cancer stem cells [55]. It has been recently reported that the disruption of nuclear complexes of CTNNB1 and HNF1A suppressed pancreatic tumor growth [56]. Wnt signaling has been widely implicated in cancer, especially colorectal cancer, in which mutation of key regulatory factors of the Wnt pathway (mainly APC and CTNNB1), was found in ninety percent of tumors, resulting in activation of the Wnt pathway [57–58]. However, the impact of Wnt signaling in PDAC is less clear. Although mutations of key Wnt pathway components are uncommon in PDAC, DNA methylation and expression status of multiple genes are involved in the regulation of Wnt pathway [59]. Nuclear localization of β-catenin is also regularly found in PDAC [60]. Inhibition of Wnt signaling using either a Wnt antagonist or a therapeutic monoclonal antibody in mice has been found to delay PDAC formation [61].

We have also noticed that some proteins mainly derived from polymorphonuclear neutrophils (PMNs), including MMP8, MMP9, MPO and PRTN3, were significantly upregulated in “short” survival patients. PMNs have received attention in the context of inflammation-driven tumorigenesis [62]. More neutrophils were found to be infiltrated in tumor cells in PDAC patients with poor survival [63, 64]. It is suggested that neutrophil-derived matrix-degrading proteases such as MMP8 and MMP9, might modulate the composition of the extracellular matrix and facilitate metastasis [65]. However, the expression of MMP8 and MMP9 can also be detected in tumor cells in patients with PDAC [66]. MMPs are also part of the apoptotic process: they cleave CDH5, PECAM1 and CDH1 during apoptosis of endothelial or epithelial cells [67]. PRTN3, also known as Myeloblastin and c-ANCA, is implicated in degradation of elastin, fibronectin, laminin, vitronectin, and collagen types I, III, and IV in *in-vitro* studies. Furthermore, PRTN3 has been shown to be involved in the degradation of extracellular matrix (ECM) proteins [68]. G12C mutation in the KRAS gene is associated significantly with an altered activity of PRTN3 in pulmonary adenocarcinomas [69]. Downregulation of PRTN3 has also been reported to inhibit proliferation and induces differentiation of promyelocyte-like leukemia cells [70].

In the light of the differential expression of several extracellular proteases, MMP8, MMP9, PRTN3 and DPP4, another protein, CLCA1, merits special mention. It is a novel self-cleaving extracellular metalloprotease [71, 72] and is a homologue of likely tumor suppressors, CLCA2 and CLCA4 [73, 74]. Low expression level of CLCA1 was observed to be linked to poor prognosis in colorectal cancer and CLCA1 itself has been proposed as a prognostic marker [75, 76]. Thus, it is an attractive hypothesis that CLCA1 has a role in tumor suppression in PDAC, either by interaction with tumor microenvironment or by proteolytic activation of yet undiscovered substrates. Another explanation of the link between CLCA1 expression and survival is the confirmed role of this protein in modulating the TMEM16A/ANO1 Ca^2+^-activated chloride channel [72, 77]. Ion channels in general, and Ca^2+^-activated chloride channels in particular are known to be involved in regulating cell proliferation, cell migration and metastasis and are believed to be important emerging cancer drug targets in cancer [78, 79], particularly in pancreatic cancer where they may be mediating interactions with the tumor microenvironment [80].

Apart from dysregulated pathways and processes, several of the proteins differentiating “long” and “short” survivors were previously noted as potential tumor markers. FAM96A, upregulated in “long” survivors, has been previously shown to regulate the iron-sulphur cluster assembly [81] and was reported to be a tumor suppressor [82]. CDH1 and CDH17 are also upregulated in “long” survivors. CDH17 is a known gastric cancer marker [83] while upregulation of CDH1 inhibits pancreatic cancer metastasis [84]. Another potential prognostic biomarker upregulated in “long” survivors in this study, LGALS4, was proposed as exocrine-like subtype PDAC marker [85]. Its homologue, LGALS1, has been previously reported to be associated with long-term survival in PDAC [15].

Another novel observation notable among proteins significantly correlated to survival is the UPF0553 protein C9orf64. This is a typical example of an interesting protein whose obscure gene symbol makes it likely to be ignored in large-scale studies [86]. In fact, C9orf64 is a protein of Q_salvage family in the Pfam database (PF10343, previously called DUF2419). Similar to DNA glycosidases and ribonucleoside hydrolases, it is involved in salvaging the micronutrient queuosine [87]. The importance of queuosine, which is involved in tRNA covalent modifications [88] is starting to be appreciated, as its roles in modulating cell proliferation are elucidated and correlation of queuosine deficiency of tRNA to severity of malignancy is revealed [89]. Thus, our results provide the first hypothesis that a link may exist between queuosine modifications and PDAC.

In conclusion, we have identified several tumor-expressed proteins that offer prognostic information in PDAC. Of note, TP53 related proteins and neutrophil-derived proteins were upregulated in PDAC patients with poor survival, supporting their potential role in tumor progression. Our results indicate that the tumor microenvironment, with an activated stroma state, is closely related to disease progression. The findings also highlight the importance of the Wnt signaling pathway. Nevertheless, there are some limitations of the present study that deserve to be mentioned. Firstly, by employing a label-free quantification, only a relative quantification was possible and the absolute upregulation or downregulation of proteins in each survival group remains unknown. Incorporating corresponding normal tissues would also be of value. Secondly, the prognostic significance of the biomarker candidates needs to be validated in larger cohorts with alternative approaches, which are more accessible in the clinic, such as immunohistochemistry and tissue microarray technology. Finally, we recommend further in-depth analysis into the mechanistic role of identified biomarker candidates in order to better understand the pathophysiological events in PDAC.

## MATERIALS AND METHODS

### Patients and samples

**Figure 9:**
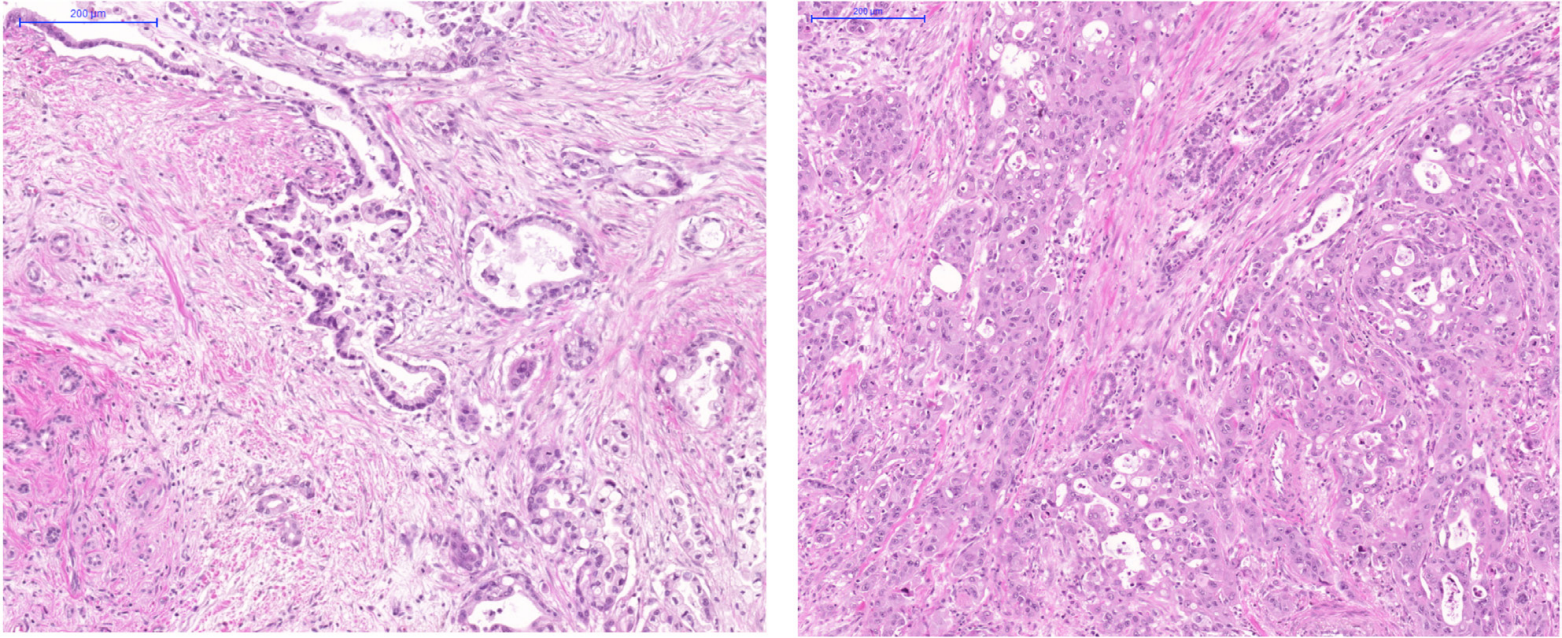
Histology images of FFPE slides from two representative cases H&E, 10x objective magnification. Left: A long survival PDAC case with histological grade 2. Notice irregular gland formation located in rich stroma. Abnormal epithel imitating normal duct epithel. Adenocarcinoma is situated in upper right part of the picture and infiltrate an atrophied pancreas parenchyma. Right: A short survival PDAC case with histological grade 3. Notice solid area of cancer structures of cells with nuclear pleomorphy and relative scanty cytoplasma. Stroma is not dominant in this picture.

Patients with surgically resectable PDAC were diagnosed and underwent surgery at the Department of Surgery, Skåne University Hospital, Lund, Sweden, between the year of 1995 and 2011.Archival FFPE tissue samples were obtained from the primary tumor and the tissue blocks were sectioned at a thickness of 10 μm. The hematoxylin-eosin staining FFPE slides from each patient were carefully reviewed by our pathologist (Figure 9). For each patient, two sections were collected in one tube, which were barcoded to be traceable and referred to their patient identities. These tubes were stored in the South Swedish Biobank, which is located in the Center of Excellence in Biological and Medical Mass Spectrometry (CEBMMS), at the Biomedical Center (BMC), Lund, Sweden. From the biobank, we retrospectively selected patients with PDAC who met the following criteria: 1) “short” survival (< 12 months) or “long” survival (> 45 months); 2) resectable disease; 3) tumors located in the head of the pancreas. Accordingly, 9 patients with PDAC with “short” survival and 10 patients with “long” survival were selected for further study. There were no significant differences in terms of pathologically confirmed lymph node metastasis, R1 resection status and use of chemotherapy between “short” and “long” survival groups. The clinical characteristics of the patients are summarized in Table 2. Ethical approval for this study was granted by the institutional review board at Lund University.

**Table 2:** Patient characteristics

	PDAC (SS)	PDAC (LS)
Sex (female/male)	3/6	7/3
Age [median (range), year]	64 (48–74)	71 (43–77)
Diabetes mellitus	5	4
Tumor location pancreas head	9	10
Tumor diameter (cm)	2.5 (1–6)	3 (2–7)
Lymph node metastasis	4	7
Staging		
IIA	5	3
IIB	4	7
R1 resection	4	3
Surgery	9	10
Adjuvant chemotherapy	5	9
Gemcitabine	3	5
5-FU	1	1
Capecitabine	0	2
Gemcitabine, 5-FU	1	0
Gemcitabine, Capecitabine	0	1
Radiotherapy	1	0
Survival (mean (SD), month)	7.3 (1.9–11.5)	59.1 (47.0–120.9)

Abbreviations: PDAC: Pancreatic ductal adenocarcinoma; SS: short survival; LS: long survival.

### Sample preparation

Two sections of FFPE tissues (10 μm) from each patient were obtained and incubated in 1mL of 1:50 diluted EnVision™ FLEX Target Retrieval Solution, High pH (Dako, Glostrup, Copenhagen, Denmark) for 10 min at 97°C, followed by centrifugation at 14,000g for 3 min and removal of the supernatant. After a repeated de-paraffinization step, the pellets were incubated in 1mL 500 mM Tris-HCl pH 8.0 at 90°C for 1.5 hours to break down cross-linking between proteins and other molecules. This was followed by centrifugation at 14,000g at 4°C for 15 min, and the supernatant was removed. For denaturation and extraction of proteins, 250 µL 6 M Guanidine-HCl in 50 mM Ammonium bicarbonate (AMBIC) was added and sonication was applied by sonication probe (Branson SLPe, Emerson Electric Co., St. Louis, MO, USA), operating with 20% amplitude, 5 min for 2 times and 20 seconds cool down period in-between on ice. After centrifugation at 14,000g for 10 min, the supernatant was stored. Protein concentration was determined by Micro BCA Protein Assay Kit (Thermo Fisher Scientific, San José, CA, USA). For each sample 150 μg proteins were diluted by AMBIC in a final volume of 180 μL and 7.5 μL of chicken lysozyme (0.02 μg/μL) was added to evaluate the variance from sample handling and instrument performance among samples. Following reduction with 3 mM DTT (1 h at 56°C) and alkylation with 15 mM iodoacetamide (30 min at 24°C in dark), the samples underwent precipitation with 1:9 volume ratio of samples to pure ethanol overnight. This was followed by centrifugation at 14,000g at 4°C for 15 min and carefully removal of the supernatant. The pellets were dissolved in 200 μL AMBIC, followed by adding 1.25 μg trypsin (Promega, Madison, WI, USA) for digestion at 37°C for 18 h. Peptide concentrations were determined by Micro BCA kit.

Exploiting strong cation exchange by Microspin column (MA SEM HIL-SCX, 10–100 μg capacity, The Nest group Inc., South Borough, MA, USA), 30 μg peptides from each sample were separated into 5 fractions by applying step-wise gradient of 20 mM, 40 mM, 60 mM, 100 mM and 500 mM KCl in 10 mM KH_2_PO4 containing 20% ACN (pH = 2.8). Each fraction underwent desalting by Ultra Microspin Silica C18 column (SUM SS18V, 3–30 μg capacity, The Nest group Inc.). Fractions were dried by centrifugal evaporator and each fraction was resuspended with 30 μL of solvent A (0.1% formic acid).

### nanoLC-MS/MS analysis (Discovery phase)

The digested peptides were loaded onto a C18 trap column (Acclaim PepMap 100 pre-column, 2 cm x 75 μm ID, 3 μm particles, 100 Å pore size, PN: 164705, Thermo Fisher Scientific) and then separated on a C18 analytical column (EASY-Spray column, 25 cm x 75 μm ID, 2 μm particles, 100 Å pore size, PN: ES802, Thermo Fisher Scientific). A flow rate of 300 nL/min and a column temperature of 35°C were applied. A nonlinear gradient was exploited using solvent A (0.1% formic acid) and solvent B (0.1% formic acid in acetonitrile). The gradient went from 7% to 26% B during the first 70 min, then increasing to 35% B during the next 20 min, followed by a raise to 90% B in 5 min, which was maintained for 15 min. The total amount of fractionated protein digest injected onto the column was estimated to be 1 μg. Fractionated samples were injected in the order of increasing salt concentrations used for elution of the peptides. To avoid carryover, each sample injection was followed by a blank injection with solvent A. Each fraction was measured for three times.

The fractionated protein digests were analysed on a Q-Exactive Plus mass spectrometer connected to an Easy-nLC 1000 pump (Thermo Fisher Scientific) with a top 10 data-dependent acquisition (DDA) method. For ionization, 1.8–2.0 kV of spray voltage and 280°C capillary temperature were used. Full MS scans were acquired with the Orbitrap mass analyser over m/z 350–1800 range with resolution of 70,000 (at m/z 200), target AGC value of 1e6 and maximum injection time of 100 ms. The ten most intense peaks with charge state >= 2 were fragmented in the HCD collision cell with normalized collision energy of 30%, and tandem mass spectra were acquired in the Orbitrap mass analyzer with resolution of 35,000 (at m/z 200), target AGC value of 1e6 and maximum injection time of 120 ms. The ion selection threshold was set to 4.2e4 and dynamic exclusion was 20 s.

### Verification by parallel reaction monitoring

Using unfractionated protein digests from each sample, a targeted proteomic method, parallel reaction monitoring (PRM) was employed to verify the differentially expressed proteins. One or two unique peptides of each protein of interest were selected. A panel of 110 peptides from 73 proteins was finally scheduled in one run to verify potentially prognostic proteins in 10 patients with “long” survival and 9 patients with “short” survival. Five peptides from chicken lysozyme and five PRTC peptides (Product no 88320, Pierce, Rockford, IL, USA) were added to the PRM panel to evaluate the experimental process. The samples were prepared in the same way as it was described previously but without SCX fractionation. The retention time, precursor m/z and charge state of peptides was referred to the prior DDA experiments. The retention times and transitions were further modified and confirmed in several preliminary PRM runs. The same LC-MS platform was applied for the PRM study. A total of 1 μg peptide was injected and the same LC parameters were used for the separation. Targeted MS^2^ mode was operated with time-scheduled acquisition of the selected peptides in +/− 5 min retention time windows. PRM scanning was performed at 17,500 resolution (AGC target 1 × 10^5^, 50 ms maximum injection time) as triggered by a scheduled inclusion list. The chromatographic peak width is 30 s. Fragmentation was performed with normalized collision energy of 27 and MS/MS scans were acquired with a resolution of 70,000 at m/z 200.

### Statistics and bioinformatics

Exploiting multidimensional protein identification technology (MudPIT), the data from 5 fractions of each sample were submitted together to Sequest HT search engine in Proteome Discoverer 1.4, being processed as one continuous input file for protein identification and quantification. The quantification of protein intensities is based on the averaged intensities of their three most abundant peptides. Uniprot Human Reviewed (released 2013/09) was referred as search database. Decoy database containing reversed version of all protein sequences were added for the monitoring of false discovery rate (FDR). For the identification of peptides, precursor and fragment mass tolerances were 10 ppm and 0.02 Da respectively. Oxidation and carbamidomethylation were taken into consideration as variable and static modifications, respectively, and one maximum missed cleavage site was allowed. Proteins were identified based on at least two peptides with high confidence (FDR < 1%). Precursor ions area detector was applied in the search engine for the quantification of peptides. Redundant proteins were automatically grouped by default. Perseus software [90] was used for the statistics. Those proteins that were detected in less than half (<5) of the samples in both groups were excluded from further analysis. To minimize the technical variance introduced by sample handling and instrument, each sample was run for three times (replicates) whereas the intensities of proteins in each replicate were normalized to its median intensity. Log 2 transformation was applied to the normalized intensities to make the data normally distributed and suitable for further statistics. Missing values were replaced from random numbers drawn from a normal distribution, which represents low abundance measurements (default setting). Using Student’s *t*-test, protein intensities were compared between two groups based on the average of log 2 transformed normalized protein intensities in each sample. Proteins were also defined as differentially expressed if detected more frequently (≥ 5 samples) in one group than in the other group. Hierarchical clustering and principal component analysis were also performed to visualize any significant differences between two groups. Skyline software was used for MS1 filtering and MS1 quantitation in the PRM study. The intensities of targeted peptide of each protein were log 2 transformed and then compared between groups by Student’s *t*-test. For those proteins having two targeted peptides, the peptide with higher intensity will be compared. The bioinformatics analysis of relationship networks between differentially expressed proteins used STRING [19] and Ingenuity Pathway Analysis (IPA, Qiagen, Inc. Redwood City, CA, USA). Assessment of overrepresented functional annotations and pathways was performed using Gene Ontology resources [91], Panther [92], Reactome [93], David [94] and IPA. In David, Panther and IPA, the whole sets of proteins detected in the study were used as analysis backgrounds.

## Abbreviations

CV: coefficient of variation
DDA: data-dependent acquisition
ECM: extracellular matrix
FDR: false discovery rate
FFPE: formalin-fixed paraffin-embedded
IPA: Ingenuity Pathway Analysis
MMPs: matrix metalloproteases
MudPIT: multidimensional protein identification technology
PC: Pancreatic cancer
PDAC: pancreatic ductal adenocarcinoma
PMNs: polymorphonuclear neutrophils
PRM: parallel reaction monitoring
PSMs: peptide-spectrum matches
